# Subjective age and retirement intentions as predictors of retirement status among 50+ adults

**DOI:** 10.3389/fpsyg.2026.1737289

**Published:** 2026-02-16

**Authors:** Antanas Kairys, Olga Zamalijeva, Raimonda Sadauskaitė, Halldór S. Guðmundsson, Anna Nyberg, Ieva Reine

**Affiliations:** 1 Institute of Psychology, Vilnius University, Vilnius, Lithuania; 2 Faculty of Social Work, University of Iceland, Reykjavík, Iceland; 3 Department of Public Health and Caring Sciences, Uppsala University, Uppsala, Sweden; 4 Statistics Unit, Rīga Stradiņš University, Riga, Latvia

**Keywords:** ageing, retirement, retirement intentions, retirement status, subjective age

## Abstract

With an aging population in Europe, it is important to understand psychological factors behind retirement decisions, since many middle-aged and older workers express a wish to retire early. Building on evidence that subjective age is embedded in identity processes and relates to retirement decision-making, this study applies an identity-based motivation perspective to address the limited empirical research on subjective age and retirement intentions interplay by testing whether subjective age moderates the extent to which retirement intentions translate into subsequent retirement behavior. Data were drawn from Waves 7 (2019/2020), 9 (2021/2022), and the COVID-19 survey (2021) of the Survey of Health, Ageing and Retirement in Europe (SHARE). The sample comprised 3,075 respondents aged 50 and older who were employed in Wave 7, received an old-age pension in Wave 9, participated in all three waves and had valid values for all variables used in the study. Retirement intentions were measured with one item, subjective age – as the difference between how old individuals feel compared to their actual age. A generalized mixed-effects analysis was conducted using R. Results showed that older subjective age and retirement intentions predicted a greater likelihood of retirement. Subjective age moderated this association: the link between feeling older and retirement was more pronounced among those with retirement intentions. These associations remained significant after controlling for demographic, socioeconomic, and health-related factors. The findings support an identity-based motivation perspective by indicating that subjective age, as an identity-related construct, may function as a moderator in the retirement intention – behavior link.

## 1 Introduction

Europe is experiencing rapid aging, with the EU’s old-age dependency ratio rising from 29% (2010) to 36% (2022) and projected to reach 59% (2070), with most of the increase occurring before 2045. These demographic changes raise concerns about sustaining a sufficient workforce across many areas, particularly in labor-intensive sectors such as healthcare (European Commission, 2024).

The pressure arising from demographic change to encourage older people to remain in the labor market longer interacts with individuals’ intentions about the timing of retirement. Retirement is not a simple shift from work to non-work, but a gradual process shaped by intentions (Topa et al., 2009) and identity processes (Bordia et al., 2020). Two main models describe this process: the two-phase model, which distinguishes between intention (decision) and action (Solem et al., 2016), and the three-phase model, which adds a stage of preferences (thoughts), emphasizing that retirement is a long-term, multi-pathway process rather than a time-bounded decision (Prothero and Beach, 1984; Topa et al., 2009). Since preferences and intentions often overlap (Topa et al., 2009), the two-phase model is conceptually more coherent, as both the preference and intention to retire or continue working reflect the same underlying process, which will be followed in this study.

Studies have shown that around half of middle-aged and older workers intent to leave the workforce upon reaching retirement age (Stynen et al., 2016), and among middle-aged and older employed individuals, nearly half likewise expressed an intention to retire as early as possible (Sadauskaite and Kairys, 2023). Such high numbers of older workers intending to leave the workforce raise questions about the possible factors behind this intention. In addition to economic and health-related factors that have been widely examined in relation to retirement decisions (e.g., Fabiani, 2024; Polanco et al., 2024), retirement intention is also shaped by how people perceive their own aging and future work capacity - making subjective age a plausible psychological driver of retirement intentions.

Subjective age “refers to how young or old individuals experience themselves to be relative to their chronological age” (Stephan et al., 2015, 1). It is associated with multiple dimensions of functioning, including physical and mental health, optimism, and hope (Aftab et al., 2022; Kotter-Grühn et al., 2015). Of particular relevance to this study, subjective age has been linked to early retirement intentions and retirement behavior (Steiber and Haas, 2023; Telitsyna et al., 2024), as well as broader economic behaviors such as retirement decisions (Ye and Post, 2020).

Possible mechanisms underlying the formation of subjective age involve identity-related and social or temporal comparison processes (Kotter-Grühn et al., 2015; Sayag and Kavé, 2022). Consequently, subjective age is sensitive to experiences of ageism and discrimination, making its study particularly relevant in later life. Perceived age discrimination is associated with feeling older, as individuals who experience age-related discrimination tend to internalize negative aging stereotypes (Stephan et al., 2015). Such discriminatory experiences also act as social stressors that impair physical and mental health, thereby further contributing to an older subjective age (Stephan et al., 2015).

It is particularly noteworthy that in the model proposed and tested by Steiber and Haas (2023), subjective age functions as a mediator between working conditions and retirement preferences, indicating that subjective age interacts with other constructs in shaping retirement-related decisions and behaviors. In the present study, however, we adopt an identity-based perspective and focus on how subjective age shapes the implementation of retirement intentions. From an identity-based motivation theoretical model (Oyserman, 2009) perspective, identity-based motivation triggers a predisposition toward certain behaviors, even when those actions lack personal utility or would not have been selected in a different situational framework. Given that subjective age is a product of identity processes, social comparison, and internalized age-related stereotypes (Kotter-Grühn et al., 2015; Sayag and Kavé, 2022; Stephan et al., 2015), there are clear theoretical reasons to expect that subjective age interacts with retirement intentions in shaping retirement behavior; however, empirical studies that explicitly test this interaction are scarce. Accordingly, we hypothesize that it may modify the way in which retirement intentions translate into retirement behavior, thus functioning as a moderator in the context of retirement behavior.

Previous studies indicated that age, gender, level of education, place of residence, marital status, health and financial situation are related to retirement behavior (Eurofound, 2012; Goll, 2020; Zaccagni et al., 2024). The proportion of individuals who continue working while receiving a pension varies across Europe (Goll, 2020). Therefore, it is important to control for the aforementioned factors in the analysis.

## 2 Objectives

The aim of the study is to examine the relationship between subjective age and retirement intentions, as well as their interaction in predicting retirement status.

## 3 Methods

### 3.1 Sample characteristics

This analysis utilized data from two regular waves [Wave 7 (W7; 2019/2020) and Wave 9 (W9; 2021/2022)] and COVID-19 telephone (Wave 9 COVID CATI - W9ca, 2021 summer) survey of the Survey of Health, Ageing and Retirement in Europe (SHARE) (Börsch-Supan et al., 2013; SHARE-ERIC, 2024a,b). For response and retention rates, see Bergmann et al. (2019). The analysis included responses from 3,075 respondents who met the following criteria: (a) were aged 50 years or older at W7; (b) were employed in W7; (c) received an old-age pension in W9; (d) participated in W7, W9, and W9ca; (e) had valid values for all variables used in the study. The W7 (baseline) interview age ranged from 51 to 92 years (*M* = 61.9, SD = 4.3). The difference between subjective and biological age ranged from −57 to 87 (*M* = −6.2, SD = 8.1). Women comprised 51.6% of the sample (*n* = 1,588). In W9, 87.0% (*n* = 2,676) of respondents identified themselves as retired; in W7, 47.5% reported that they would like to retire as soon as possible; and in W7, 21.9% (*n* = 672) were receiving an old-age pension. Respondents were from 27 countries (Europe and Israel), with the largest number from Estonia (457) and the smallest from Cyprus (21). A total of 1,023 respondents (33.3%) had tertiary education; 1,237 lived in a big city, suburbs of a big city, or a large town; and 2,427 (78.9%) had a partner in the household. The median annual income per household member was €8,000. In addition, 1,116 respondents (36.3%) had two or more chronic diseases, and 974 (31.7%) reported long-standing activity limitations due to health problems.

### 3.2 Measures

#### 3.2.1 Self-reported employment status

Self-reported employment status was used both as a filtering variable (W7) and as a dependent variable (W9). In both cases, it was based on responses to the question: “In general, which of the following best describes your current employment situation?” Only respondents who in W7 selected “Employed or self-employed (including working for family business)” were included in the study. The dependent variable in the analysis was the response to the same question in W9, coded as 0 for “Employed or self-employed (including working for family business)” and 1 for “Retired.” Respondents who selected other options, such as “Permanently sick or disabled,” were excluded from the analysis.

#### 3.2.2 Subjective age

Subjective age was assessed using a question included in W9ca: “Many people feel older or younger than they actually are. What age do you currently feel?” Respondents were asked to report their subjective age in years. In the analysis, the measure used was not the reported subjective age itself but the difference between subjective and chronological age (DBSCA), thereby reflecting whether respondents perceived themselves as younger, older, or the same age as their chronological age. Due to high kurtosis and the presence of extreme values, this variable was winsorized at the 1% level.

#### 3.2.3 Retirement intentions

Retirement intentions were measured in W7 using the question: “Thinking about your present job, would you like to retire as early as you can from this job?” Responses were coded as 1 = “yes” and 0 = “no.”

#### 3.2.4 Control variables

Control variables included demographic variables (age, gender, level of education, place of residence, and whether the respondent had a partner in the household), socioeconomic variables (receipt of a pension in W7 and income), and health-related variables (number of chronic diseases and the GALI index). Based on the most common definition, multimorbidity was operationalized as a binary variable indicating whether the respondent reported having two or more chronic conditions (Johnston et al., 2019). GALI is a single-item indicator measuring long-standing (6 months or longer) activity limitations due to general health problems (Robine and Jagger, 2003); it was recoded into a binary variable. Gender, level of education, place of residence, presence of a partner in the household, and pension receipt were also recoded into binary variables (information on coding is provided in Table 1). The income measure was taken from the first imputation, following the procedure described by Gruber (2019). In countries where income was reported in a currency other than euros, values were converted to euros. Household income was then divided by the number of household members. Due to high skewness, a 1% winsorization was applied, after which the income variable was log-transformed. Data for all control variables were derived from W7.

**TABLE 1:** Means, standard deviations and correlations among study variables.

Variable	*M* (SD) or *N* (%)	1	2	3	4	5	6	7	8	9	10	11
1. Retirement intentions^a^	1,462 (47.54%)	–	–	–	–	–	–	–	–	–	–	–
2. Number of chronic diseases^a^	1,116 (36.29%)	0.038*	–	–	–	–	–	–	–	–	–	–
3. GALI index^a^	974 (31.67%)	0.047**	0.332***	–	–	–	–	–	–	–	–	–
4. Gender^a^	1,487 (48.36%)	−0.014	−0.013	−0.052**	–	–	–	–	–	–	–	–
5. Age^a^	61.89 (4.26)	−0.280***	0.094***	0.030	0.130***	–	–	–	–	–	–	–
6. Place of residence^a^	1,237 (40.23%)	−0.040*	0.048**	−0.021	−0.004	0.113***	–	–	–	–	–	–
7. Level of education^a^	1,023 (33.27%)	−0.139***	−0.003	−0.025	−0.033	0.117***	0.127***	–	–	–	–	–
8. Income per household member (winsorized, log)^a^	8.67 (1.48)	−0.084***	0.001	−0.003	−0.023	0.110***	0.002	0.162***	–	–	–	–
9. Partner in household^a^	2,427 (88.68%)	0.053**	−0.043*	−0.060***	0.197***	−0.101***	−0.072***	−0.006	−0.044*	–	–	–
10. Difference between subjective and chronological age (winsorized, centered)^b^	0.00 (7.45)	0.075***	0.059***	0.065***	0.034	−0.084***	−0.026	−0.040*	−0.032	0.042*	–	–
11. Receives pension in W7^a^	672 (21.85)	−0.212***	0.048**	0.053**	0.016	0.571***	0.052**	0.037*	0.055**	−0.099***	−0.061***	–
12. Self-reported retirement status^c^	2,676 (87.02%)	0.184***	0.036*	0.017	−0.006	−0.115***	−0.038*	−0.052**	−0.053**	0.038*	0.113***	−0.156***

*M*(SD) presented for continuous variables, *N*(%) for dichotomous. *N*(%) presented for “1” coded category. number of observations: 3,075. Coding of dichotomous variables: retirement intentions: 1 wants to retire; 0, no retirement intentions; Number of chronic diseases: 1–2 or more; 0, 0 or 1 chronic disease; GALI: 1, has activities limitations; 0, no limitations; Gender: 1, male; 0, female; Place of residence: 1, big city, suburbs of big city or large town; 0, small town, village or rural area; Level of education: 1, tertiary education; 0, less than tertiary education; Partner in household: 1, has partner in household; 0, no partner in household; Receives pension in W7: 1, receives old age pension in Wave 7; 0, don’t receives old age pension in Wave 7.

***P < 0.001,

**p < 0.01,

*p < 0.05. ^a^Measured in W7. ^b^Measured in W9ca. ^c^Measured in W9.

### 3.3 Statistical analysis

Given that the data were clustered, a generalized mixed-effects analysis with a dichotomous dependent variable was conducted using the glmer function (lme4 library) in R. A random intercept model was specified (country as grouping variable)^1^. While the model accounts for country-level clustering using random intercepts, it does not aim to explain specific cross-national differences, which remain beyond the scope of this analysis. Additionally, correlations among the study variables were computed and simple slopes analysis was performed (using library interactions).

## 4 Results

Means, standard deviations and correlations among study variables are presented in Table 1.

When examining the data presented in Table 1, it can be observed that self-reported retirement status correlates with all other study variables except for the GALI index and gender. However, these results are based on data aggregated across all countries, and therefore the country effect is not controlled for. The null generalized mixed-effects model, which included only a random intercept for country, showed that 25% of the variance in retirement status (ICC = 0.25) could be explained by differences between countries, indicating a substantial country-level effect. Therefore, subsequent analyses were conducted using a generalized mixed-effects model with random intercepts (Table 2). Due to the limited number of countries (*n* = 27), a random slopes and intercepts model was not tested.

**TABLE 2:** Summary table of mixed effects model predicting self–reported retirement status in Wave 9.

Variable	Coefficient	OR	CI OR	*z*
Intercept	−0.29	0.75	[0.05–10.89]	−0.21
**Control variables**				
Gender (1 – male)	−0.26	0.77	[0.60–0.98]	−2.14*
Age	0.05	1.05	[1.02–1.09]	2.86**
Place of residence (1 – big city, suburbs of big city or large town)	−0.11	0.89	[0.70–1.14]	−0.91
Level of education (1 – tertiary education)	−0.20	0.82	[0.64–1.05]	0.12
Partner in household (1 – has partner in household)	0.05	1.05	[0.79–1.39]	0.33
Income per household member (winsorized, log)	−0.06	0.94	[0.78–1.13]	0.49
Receives pension in W7 (1 – receives)	−0.34	0.71	[0.52–0.98]	−2.06*
Number of chronic diseases (1 – 2 + diseases)	0.28	1.32	[1.02–1.72]	2.12*
GALI index (1 – has limitations)	0.09	1.09	[0.83–1.43]	0.64
**Main variables**				
Retirement intentions (1 – wants to retire)	0.95	2.58	[1.95–3.41]	6.60***
Difference between subjective and chronological age (winsorized, centered)	0.02	1.02	[1.01–1.04]	2.71**
Interaction: retirement intentions X DBSCA	0.04	1.04	[1.01–1.07]	2.33*
**Random effect**				
	Variance	SD		
Country (27 countries included)	1.01	1.00	–	–

Dependent variable: self – reported retirement status (1, retired; 0, working); number of observations: 3,075. OR, odds ratio; CI, confidence intervals. The full model with all predictors and a random intercept for country showed substantially better fit than the null model with only random intercept for country (AIC = 2006.0 vs. 2080.1; BIC = 2090.4 vs. 2092.2), and the likelihood-ratio test was significant, χ^2^(12) = 98.15, *p* < 0.001. Nakagawa’s pseudo-R^2^ indicated R^2^m = 0.095 (fixed effects) and R^2^c = 0.307 (fixed + random effects).

***P < 0.001,

**p < 0.01,

*p < 0.05.

**FIGURE 1:**
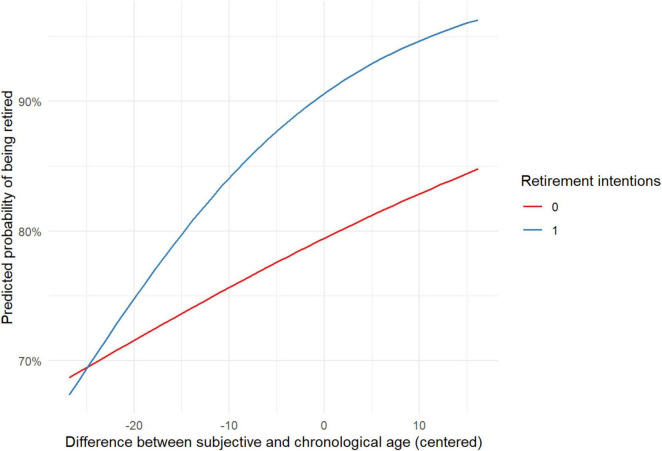
Interaction between difference of subjective and chronological age and retirement intentions. Retirement intentions: 1, wants to retire; 0, no retirement intentions.

The results presented in Table 2 indicate that among the control variables, age and having 2+ chronic conditions were associated with a higher likelihood of being retired in W9, whereas receiving a pension in W7 was associated with a lower likelihood of being retired. Both main study variables – DBSCA and retirement intentions – were related to self-reported retirement status: having retirement intentions and feeling older relative to one’s chronological age were associated with a higher likelihood of being retired. An interaction between DBSCA and retirement intentions was also found and visualized (Figure 1).

Both Figure 1 and the simple slope analysis show that feeling older than one’s chronological age is associated with a higher likelihood of being retired, regardless of whether retirement intentions are present or not. However, the effect is stronger among those with retirement intentions (*b* = 0.06, *z* = 4.46, *p* < 0.001) compared to those without such intentions (*b* = 0.02, *z* = 2.71, *p* < 0.05).

## 5 Discussion

This study, based on a representative sample of individuals aged 50 and over from 27 countries, revealed important associations between retirement intentions, subjective age, and retirement status, while also indicating a substantial country-level effect. Among the contextual factors, age and multimorbidity increased the likelihood of being retired, while receiving a pension in W7 decreased it. Both subjective age and retirement intentions predicted retirement status, and their interaction showed that feeling older was more strongly associated with being retired among those who had retirement intentions.

The principal finding of this research is that feeling older than one’s chronological age not only predicts the likelihood of being retired, but also amplifies the association between retirement intentions and subsequent retirement behavior. The result that feeling older predicts the probability of being retired is in line with previous research showing that subjective age is related to retirement intentions and retirement decisions (Steiber and Haas, 2023; Telitsyna et al., 2024; Ye and Post, 2020). The finding that subjective age operates as a moderator is, however, novel. We interpret this effect within the identity-based motivation framework (Oyserman, 2009): subjective age, as an age-related identity construct, appears to strengthen retirement intentions as a predisposition toward retirement behavior. In other words, feeling older than one’s chronological age – potentially linked to experiences of ageism (Stephan et al., 2015) – is associated with a higher likelihood of being retired irrespective of retirement intentions. However, this retirement-favoring association of subjective age is more pronounced when individuals already report retirement intentions.

Official statistics (Eurostat, 2025) show substantial cross-national differences in the number of people working after retirement in Europe; therefore, it is not surprising that our analysis also revealed significant country-level variation. Different countries not only have different retirement schemes, but these schemes are also subject to change over time within a particular country. Moreover, country-level regulations may interact with sector and work type indicators, for instance, in some countries people working in the public sector may have strict statutory retirement age, while private sector may be more flexible in that regard, especially if they want to retain skilled and experienced employees. It is also possible that maintaining non-manual labor, which is less reliant on physical strength and endurance, could be easier in later life. Thus, the topic of the role of subjective age and retirement intentions in understanding actual retirement in different cultural and work specific contexts, has yet a lot to offer, therefore, it is worthwhile to include country-level variables in subsequent analyses.

## 6 Limitations and future research

Despite being based on cross-national sample and longitudinal design, this research could not avoid limitations. Although this analysis uses a probability sample, it is still possible that some sample selection bias was not fully eliminated Our analysis revealed pronounced cross-country differences in actual retirement; however, country-level variables were not included in the model, as the analysis primarily focused on examining subjective age and retirement intentions. Therefore, future research should consider incorporating country-level indicators such as GDP per capita, statutory retirement age, and unemployment rate – and their interactions with individual–level variables. Another limitation of this study is that subjective age was measured two years after the main study variables (retirement intentions and other independent variables in W7 → subjective age in W9ca → retirement status in W9). This timing may have introduced additional bias, as subjective age could reflect changes that occurred in respondents’ lives during the 2-year interval. Therefore, when possible, it is recommended to measure all variables concurrently or repeatedly (thereby enabling a longitudinal research design). Finally, it is important to note that the study covered the period of the COVID-19 pandemic; therefore, participants may have experienced additional health challenges or work disruptions related to the illness or pandemic-related restrictions. Consequently, it would be important to replicate the study’s findings in a period unaffected by the pandemic.

## 7 Conclusion

Subjective age and retirement intentions predicted future retirement status. Moreover, the interaction of the main predictors showed that feeling older than their actual age increased the chances of retiring among employed middle aged and older adults who had retirement intentions. Female gender, older age, not receiving pension, and presence of multiple chronic conditions was also associated with greater likelihood of retirement.

## Data Availability

Publicly available datasets were analyzed in this study. This data can be found here: https://share-eric.eu/data/data-access.

## Footnotes

1 Specification of the model: Self-reported employment status ∼ retirement intentions + DBSCA + GALI + gender + age + level of education + place of residence + partner in the household + income + receipt of a pension in W7 + retirement intentions * DBSCA + (1 | country).
